# Supplementary material for: Protocol for an umbrella review of systematic reviews evaluating the efficacy of digital health solutions in supporting adult cancer survivorship care
Source: PLoS One. 2025 May 27;20(5):e0322100. doi: 10.1371/journal.pone.0322100 (PMC12111579; doi:10.1371/journal.pone.0322100)
Supplement: S1 Table — (DOCX) [file pone.0322100.s002.docx]

# **S2 Table: Detailed eligibility criteria outlined according to PICO framework(1)**

| PICO | **Inclusion criteria** | **Exclusion criteria** |
| --- | --- | --- |
| Population | Adults, as defined by the included study authors, as being diagnosed with cancer and can be living with, through or beyond cancer.  Adults who have had an experience of cancer in their childhood.  Papers that deal with adolescents and young adults only if the results are stratified or differentiated for those categorized as adults by the authors.  Papers that also deal with carers or healthcare professionals only if the results are stratified or differentiated for patients categorized as adults by the authors. | Data and studies pertaining to paediatrics, caregivers, health care professionals, family members only or where results cannot be differentiated from patient outcome data. |
| Intervention/ variable of interest | Patient-oriented interventions.  Digital health solutions also termed digital health interventions or innovations or tools used to support the implementation of adult cancer survivorship care. | Studies that do not specify a digital health solution that is patient orientated.  Studies that specify a digital health solution that is family/carer orientated.  Decision support systems or digital health solutions specifically targeted at health care professionals.  Digital health solutions offered without the overt oversight or governance of health care professionals.  Digital health solutions provided without an explicit reference to an underpinning evidence base. |
| Comparison | As reported by included studies | No specific eligibility criteria applied |
| Outcomes | **Primary outcomes**:  Indicators from the following domains described in Quality of Cancer Survivorship Framework(2) (Table 1)   - Surveillance and management of physical effects - Surveillance and management of psychosocial effects     **Secondary outcomes**: (Table 2)   - Prevention and surveillance of new cancer/ recurring cancers - Surveillance and Management of Chronic Medical Conditions - Health Promotion and Disease Prevention     Experiences associated with these outcomes are also of interest. | Focused on satisfaction, acceptability, feasibility of the intervention.  Expectations of outcomes. |
| Design | Systematic reviews +/- meta analysis, meta summary, meta synthesis or other summative synthesis methodology.  Systematic reviews incorporating **only** experimental studies (randomised controlled trials or non-randomised studies) with a clear aim to assess the **efficacy or effectiveness** of the digital health solution(s) in terms of the named outcomes in this review will be included. Such non-randomised designs can include quasi-experimental designs, uncontrolled before and after studies, controlled before and after studies where there is the explicit examination of the effectiveness of a digital health solution(s).    Authors felt that restrictions to inclusion of randomised designs only would give an incomplete picture. | Scoping reviews, narrative reviews. Umbrella reviews.  Reviews where it is not clearly stated by the authors to be a “systematic review”.  A systematic review having a mixture of or only incorporating studies with feasibility, acceptability, satisfaction, pilot study aims will be excluded.  A systematic review having a mixture of experimental and other non-experimental designs e.g. observational studies, qualitative studies. |
| Publication types and other limits | **Publication types:** peer-reviewed systematic reviews +/- meta-analysis, +/- meta-synthesis, +/- meta-summary. Systematic reviews published within identified databases.    **Setting:** reviews incorporating studies conducted in any setting --local, regional, national or international level.    **Limits**: publications from after Jan 1^st^, 2013 to date of search.  No language limits applied. | **Publication types:**  opinion pieces, Government-level or health system level policy, papers, thesis, grey literature, conference abstracts, protocols. |

## References

1. Schardt C, Adams MB, Owens T, Keitz S, Fontelo P. Utilization of the PICO framework to improve searching PubMed for clinical questions. BMC Med Inform Decis Mak [Internet]. 2007 Dec [cited 2024 Jan 8];7(1):16. Available from: https://bmcmedinformdecismak.biomedcentral.com/articles/10.1186/1472-6947-7-16
