# Supplementary material for: Protocol for an umbrella review of systematic reviews evaluating the efficacy of digital health solutions in supporting adult cancer survivorship care
Source: PLoS One. 2025 May 27;20(5):e0322100. doi: 10.1371/journal.pone.0322100 (PMC12111579; doi:10.1371/journal.pone.0322100)
Supplement: S2 Table — (DOCX) [file pone.0322100.s003.docx]

**S3 Table. Definitions of digital health solutions**

| **Author (year)**  **Country** | **Definitions of digital health solutions** |
| --- | --- |
| EU 2018 Communication on Digital Health and Care (1)  EU | According to the EU commission digital health and care refers “*to tools and services that use information and communication technologies (ICTs) to improve prevention, diagnosis, treatment, monitoring and management of health-related issues and to monitor and manage lifestyle-habits that impact health*  *... improve access to care and the quality of that care …and increase the overall efficiency of the health sector”*([European Commission](https://health.ec.europa.eu/ehealth-digital-health-and-care/overview_en)). These can include mobile health (mHealth), health information technology (IT), telehealth, telemedicine, platforms, apps, wearable devices, sensors, and the use of digital solutions to support personalised medicine and healthcare. |
| Birnbaum et al.,  (2015) (2)  USA | “May help patients self-monitor; encourage behavior change; improve comprehension of diagnoses and discharge plans; and permit dynamic interchanges between patients, their healthcare data, and clinicians. Digital health tools have been hailed as easily disseminated, low-resource solutions to help patients take ownership of their healthcare journey” pg. 754 |
| Fredrix et al.,  (2019) (3)  Belgium | “the use of information and communication technologies to treat patients and convey healthy lifestyles (primary prevention), conduct research, educate healthcare professionals, track diseases and monitor public health as an update of the previous definition of e-health. Digital health is used as a general term, encompassing e-learning, remote monitoring (i.e. telemonitoring), structured telephone support, telerehabilitation, teleconsultation and m-health apps” pg.1167 |
| Klucken et al.,  (2018) (4)  Germany | “data-driven personalized decision support that is based on a combination of multimodal data sources, including evidence-based medical knowledge (e.g., clinical guidelines), personal disease profiles (including genetic determinants of disease progression and treatment response), insights into individual disease trajectories (thereby defining subgroups of patients) and individual patients’ needs.” Pg.S85 |
| Kostkova  (2015) (5)  England | “the use of information and communications technology to improve human health, healthcare services and wellness for individuals across populations” pg.1 |
| Sharma et al.,  (2018) (6)  USA | “using digital information, data, and communication technologies to collect, share, and analyze health information for purposes of improving patient health and health care delivery” pg. 2680 |

## References

1. EU Commision’s 2018 Communication on Digital Health and Care [Internet]. 2018. Available from: https://health.ec.europa.eu/ehealth-digital-health-and-care/overview_en

2. Birnbaum F, Lewis D, Rosen RK, Ranney ML. Patient Engagement and the Design of Digital Health. Miner J, editor. Academic Emergency Medicine [Internet]. 2015 Jun [cited 2024 Jan 8];22(6):754–6. Available from: https://onlinelibrary.wiley.com/doi/10.1111/acem.12692

3. Frederix I, Caiani EG, Dendale P, Anker S, Bax J, Böhm A, et al. ESC e-Cardiology Working Group Position Paper: Overcoming challenges in digital health implementation in cardiovascular medicine. Eur J Prev Cardiolog [Internet]. 2019 Jul [cited 2024 Jan 8];26(11):1166–77. Available from: https://academic.oup.com/eurjpc/article/26/11/1166-1177/5925151

4. Klucken J, Krüger R, Schmidt P, Bloem BR. Management of Parkinson’s Disease 20 Years from Now: Towards Digital Health Pathways. Brundin P, Langston JW, Bloem BR, editors. JPD [Internet]. 2018 Dec 18 [cited 2024 Jan 8];8(s1):S85–94. Available from: https://www.medra.org/servlet/aliasResolver?alias=iospress&doi=10.3233/JPD-181519

5. Kostkova P. Grand Challenges in Digital Health. Front Public Health [Internet]. 2015 May 5 [cited 2024 Jan 8];3. Available from: http://www.frontiersin.org/Digital_Health/10.3389/fpubh.2015.00134/full

6. Sharma A, Harrington RA, McClellan MB, Turakhia MP, Eapen ZJ, Steinhubl S, et al. Using Digital Health Technology to Better Generate Evidence and Deliver Evidence-Based Care. Journal of the American College of Cardiology [Internet]. 2018 Jun [cited 2024 Jan 9];71(23):2680–90. Available from: https://linkinghub.elsevier.com/retrieve/pii/S0735109718344139
