# Supplementary material for: Protocol for an umbrella review of systematic reviews evaluating the efficacy of digital health solutions in supporting adult cancer survivorship care
Source: PLoS One. 2025 May 27;20(5):e0322100. doi: 10.1371/journal.pone.0322100 (PMC12111579; doi:10.1371/journal.pone.0322100)
Supplement: S3 Table — A. Expanded outcome domains adapted from the Quality of Cancer Survivorship Care Framework including associated outcomes for each domain. B. Expanded Secondary Outcomes, adapted from the Quality of Cancer Survivorship Care Framework including associated outcomes. (DOCX) [file pone.0322100.s004.docx]

**S4A Table. Expanded outcome domains adapted from the Quality of Cancer Survivorship Care Framework (1) including associated outcomes for each domain**

| Domain | Associated outcomes |
| --- | --- |
| Prevention and Surveillance for Recurrence and New Cancers | - Assessment of risk predisposition - Referral and receipt of recommended genetics evaluation - Recommendation for adjuvant and/or risk-reducing strategies - Assessment of adherence with recommended adjuvant and/or risk-reducing strategies - Clinical surveillance visits recommended and completed per guidelines - Laboratory surveillance testing recommended and completed per guidelines - Imaging surveillance recommended and completed per guidelines |
| Surveillance and Management of Physical Effects | - Physical effects - Visual - Hearing - Oral/dental - Ear/nose/throat - Endocrine - Cardiac - Pulmonary - Gastrointestinal - Hepatic - Genitourinary - Immunological - Male genital - Gynaecological - Musculoskeletal - Dermatological - Neurological - Neurocognitive - Vasomotor - Vascular - Body composition - Frailty - Reduced exercise tolerance - Overall burden of physical symptoms - Referral and receipt of recommended evaluation - Recommendation and receipt of appropriate treatment - Recommendation for risk-reducing strategies (e.g., weight loss, exercise, pharmacological treatment) - Assessment of adherence to recommended treatment and/or risk-reducing strategies - Reassessment of symptoms and/or conditions at defined intervals and/or treatment phase |
| Surveillance and Management of Psychosocial Effects | **Psychological**   - Fatigue - Stress - Post-traumatic stress - Posttraumatic growth - Distress - Anxiety - Fear of recurrences - Sleep disturbance - Coping - Worry - Illness intrusiveness - Cognitive changes - Educational problems - Social withdrawal   **Financial and/or employment**   - Financial toxicity - Underemployment, unemployment - Return to work - Work productivity - School productivity - Insurance status   **Interpersonal**   - Sexuality and/or intimacy - Fertility - Family and/or caregiver relationships   Other   - Recommended evaluation provided - Treatment provided (e.g., medication, therapy, exercise) - Assessment of adherence to treatment completed |

**S4B Table. Expanded Secondary Outcomes, adapted from the Quality of Cancer Survivorship Care Framework (1) including associated outcomes**

| **Domain** | **Associated outcomes** |
| --- | --- |
| Surveillance and Management of Chronic Medical Conditions | - Evaluation and treatment of noncancer medical conditions using disease-specific indicators - Medication reconciliation |
| Health Promotion and Disease Prevention | - Prevention-focused visits and testing - Age- and gender-appropriate cancer screening, recommendation, referral, and receipt of screening - Assessment of lifestyle behaviours, referral, and treatment - Assessment of weight management, referral, and treatment - Vaccination advice and assessment of vaccination rates - Screening for exposure to infectious exposures |

## References

1. Nekhlyudov L, Mollica MA, Jacobsen PB, Mayer DK, Shulman LN, Geiger AM. Developing a Quality of Cancer Survivorship Care Framework: Implications for Clinical Care, Research, and Policy. JNCI: Journal of the National Cancer Institute [Internet]. 2019 Nov 1 [cited 2024 Jan 4];111(11):1120–30. Available from: https://academic.oup.com/jnci/article/111/11/1120/5490202
