# Supplementary material for: Protocol for an umbrella review of systematic reviews evaluating the efficacy of digital health solutions in supporting adult cancer survivorship care
Source: PLoS One. 2025 May 27;20(5):e0322100. doi: 10.1371/journal.pone.0322100 (PMC12111579; doi:10.1371/journal.pone.0322100)
Supplement: S4 Table — Detailed CINAHL sample search. (DOCX) [file pone.0322100.s005.docx]

**S5 Table. Detailed CINAHL sample search**

| Database | Search strings (combined for the table) |
| --- | --- |
| CINAHL | [Title/Abstract search]  **#1**  cancer OR cancer* OR “oncolog*” OR tumour* OR tumor* OR neoplas* OR carcinoma OR malignan* OR Haematolog* OR Hematolog* OR lymphoma OR Leukemia OR Leukemia OR Leukaemia OR sarcoma OR myeloma*    **#2**  “Seasons of survivorship" OR “Supportive care” OR survivorship OR “living with and beyond” OR “living with, through and beyond” OR palliat* OR "psycho-oncology" OR support* N3 care OR survivor* N3 care    **#3**  digital* OR mobile OR apps OR “mobile app” OR application OR apps OR android OR iPhone OR “mobile appl*” OR “online tool” OR web OR internet OR computer OR laptop OR email OR e-mail OR “social network” OR “social media” OR Facebook  OR platform OR “serious game” OR virtual OR remote OR “messaging service” OR “mobile health” OR “digital health intervention” OR “digital health technology” OR “digital health solution” OR telehealth OR telemedicine OR “e-health” OR “e health” OR ehealth OR mhealth OR “m-health” OR AI OR “artificial intelligence” OR "virtual realit*"  OR "augmented realit*" OR "mixed realit*" OR elearn* OR “e-learn*” OR robot* OR informatic* OR wearabl* OR portabl* OR "smart watch" OR smartwatch OR "smart device" OR smartphone OR "smart phone" OR fitbit OR "fitness tracker"  OR ipad OR "text messag*" OR sms      **#4 Systematic Reviews**  “systematic review*” OR “systematic literature review” OR meta-analys* OR metaanalys* OR “meta analys*" OR meta-synthes* OR metasynthes* OR “meta synthes*” OR metasummar* OR “meta summar*” OR “meta narrative” OR “network meta-analys*"  OR systematic N3 review |
