# Supplementary material for: Protocol for an umbrella review of systematic reviews evaluating the efficacy of digital health solutions in supporting adult cancer survivorship care
Source: PLoS One. 2025 May 27;20(5):e0322100. doi: 10.1371/journal.pone.0322100 (PMC12111579; doi:10.1371/journal.pone.0322100)
Supplement: S5 Table — (DOCX) [file pone.0322100.s006.docx]

S6 Table: Categories of digital health solutions, adapted from Lupton’s (2014) typology of digital health technologies(7).

| **Digital health solutions** | **Further details** |
| --- | --- |
| Telemedicine and telehealth | Medical consultations, clinical diagnosis and healthcare delivery (e.g., appointments, counselling and support services) offered remotely via digital technologies |
| Medical education, training and exchange of information between doctors and other healthcare providers using digital technologies |  |
| Digital diagnostic, risk-assessment and decision-making technologies | Apps, online tools and add-on technologies to smartphones for use by doctors |
| Digitised devices for delivering medicine or regulating/enhancing bodily functions | Cochlear implants, cardiac monitors, insulin pumps, digital pills and so on |
| Health informatics | ICT used within healthcare including electronic patient records and other online health information, triage and appointment booking systems |
| Digital health promotion | Health education messages via digital technologies |
| Biometric tracking, patient self-care and monitoring devices | Apps, smartphones, smart objects and wearable technologies for monitoring and tracking bodily functions and activities |
| Dedicated platforms for exchange of information | Patient blogs, social media sites, apps |
| Digital health games | Console, online and app games designed for fitness, tracking biometrics, health promotion and health education |

References:

7. Lupton D. Critical Perspectives on Digital Health Technologies. Sociology Compass [Internet]. 2014 Dec [cited 2024 Jan 8];8(12):1344–59. Available from: https://compass.onlinelibrary.wiley.com/doi/10.1111/soc4.12226
