# Supplementary material for: Protocol for an umbrella review of systematic reviews evaluating the efficacy of digital health solutions in supporting adult cancer survivorship care
Source: PLoS One. 2025 May 27;20(5):e0322100. doi: 10.1371/journal.pone.0322100 (PMC12111579; doi:10.1371/journal.pone.0322100)
Supplement: Attachment — Submitted filename: PLOS ONE review.docx [file pone.0322100.s007.docx]

**Reviewer Report**

Manuscript number: PONE-D-24-26581

Manuscript title: Protocol for an umbrella review of systematic reviews evaluating the efficacy of digital health solutions in supporting adult cancer survivorship care.

Decision: Minor revision

Comments:

The authors are to be commended for designing this umbrella review which plans to synthesize the existing data for digital health solutions for cancer survivorship and identify gaps for future research. The methodology is well thought out and involves a comprehensive and robust analysis of systematic reviews.

I have a two minor comments for the authors to consider:

Patients with early cancers have different supportive care needs compared to those with metastatic cancers. While the definition of “cancer survivor” refer to all patients regardless of stage and the domains of the Quality of Cancer Survivorship Care Framework apply to both groups of patients, it is relevant to categorise which interventions are for those with early cancers, and which are those for advanced or metastatic cancers. Please clarify if there is any plan to make this categorization in the analysis. If there is, the authors are suggested to provide definitions of early versus advanced and/or metastatic cancer for consistency in the categorization.

Unlike survival metrics, analysis of the effectiveness of supportive care measures may have significant variability or heterogeneity. The authors are suggested to provide a summary of all the study endpoints that have been analysed in the systematic reviews and meta-analyses, and evaluate whether they correlated with cancer survival or quality of life. This may help future clinical trials on digital health solutions select appropriate and meaningful outcome measures.
